# Antibacterial Activity of Honey against Methicillin-Resistant *Staphylococcus aureus*: A Laboratory-Based Experimental Study

**DOI:** 10.1155/2019/7686130

**Published:** 2019-04-03

**Authors:** Mohammedaman Mama, Teklu Teshome, Jafer Detamo

**Affiliations:** ^1^Madda Walabu University, Goba Referral Hospital, Department of Medical Laboratory Science, Goba, Ethiopia; ^2^Arba Minch University, College of Medicine and Health Sciences, Department of Biomedical Sciences, Arba Minch, Ethiopia; ^3^Arba Minch University, College of Medicine and Health Sciences, Department of Pharmacy, Arba Minch, Ethiopia

## Abstract

**Background:**

Antimicrobial drug resistance is one of the serious issues this world is facing nowadays, and increased cost of searching for effective antimicrobial agents and the decreased rate of new drug discovery have made the situation increasingly worrisome.

**Objective:**

The aim of this study is to determine in vitro antibacterial activity of honey against methicillin-resistant *Staphylococcus aureus* isolates from wound infection.

**Methods:**

An experimental study was conducted from May to November 2017. Methicillin resistance was detected using cefoxitin (30 *μ*g) and oxacillin (1 *μ*g) antibiotic discs. Different concentrations of honey (25–100% v/v) were tested against each type of clinical isolates obtained from wound infection. A preliminary sensitivity test was done to all types of honey by using disk diffusion while minimum inhibitory concentration and minimum bactericidal concentration were determined for the most potent honey by the broth dilution technique. All statistical analysis was performed by using Statistical Package for the Social Sciences version 20.

**Results:**

In this study, 36 bacterial isolates were recovered from 50 specimens, showing an isolation rate of 72%. The predominant bacteria isolated from the infected wounds were *Staphylococcus aureus* (15, 41.7%). Among identified *Staphylococcus aureus*, methicillin resistance accounts for 10 isolates (27.8%). All isolates showed a high frequency of resistance to tetracycline. Four collected honey varieties exhibited antibacterial activity, while the strongest inhibitory activity was demonstrated by honey-2 at 75% v/v. The mean MIC and MBC of honey-2 ranged from 9.38 to 37.5% v/v.

**Conclusions:**

Tested honey has both a bacteriostatic and bactericidal activity. Among the tested honey, “honey-2” had high antibacterial potency than others.

## 1. Introduction

Inadequate wound management compounded with secondary infection is still remaining a major public health problem in developing countries. Therefore, wound management has remained as research focus so far. Because of increased drug resistance, the interest in using alternative therapies and natural remedies in wound management has rapidly increased [1, 2].

Despite the enormous advance in health care made during the last half-century, infectious diseases still account for 25% of mortality worldwide and 45% in low-income countries. Among these, pathogenic and antibiotic-resistant bacteria pose a very serious threat to public health which makes them the main cause of mortality and morbidity in hospitals and community [3–7].

Anti-infective drugs (antimicrobial agents) are critically important in reducing the global burden of infectious diseases [8, 9]. The occurrence of drug-resistant microorganisms diminished the development of antibiotics, and few pharmaceutical companies remain active in this area, posing a big challenge in this world [10]. Hence, the failure of these antibiotics has resulted for a man to search for more effective sources of natural products from plants and others [11, 12].

The antibacterial activity of honey was first recognized in 1892; however, it has a limited use in modern medicine due to lack of scientific support [13]. Honey is the nectar collected from flowers by bees. It contains 15% to 20% water and 80% to 85% sugar. The remainder of the honey is made up of proteins, enzymes, and nonessential amino acids [14–16]. Several properties of honey like enzymes are responsible for its bactericidal effect and wound healing. Glucose oxidase which changes glucose to gluconolactone and then to hydrogen peroxide is one of the enzymes found in honey. The release of H_2_O_2_ is slow and continuous for a constant antibacterial effect, successfully eliminating microorganisms but dilute enough not to damage host tissue [17–19].

Honey having acidic pH of 3.2–4.5 is used to inhibit many pathogenic organisms and increased wound healing process through epithelization [17, 20, 21]. Honey is also one of the supersaturated solutions that inhibit bacterial growth primarily due to this high osmolarity [22].

The World Health Organization (WHO) has described alternative medicines as a cheap way to achieve total health care coverage of the world's population and has encouraged the rational use of plant-based alternative medicines by member states [23]. In Ethiopia, about 80% of the total population relies on traditional remedies as a primary source of health care [24]. Hence, the purpose of this study was to evaluate antibacterial activity (bacteriostatic and bactericidal effects) of honey against MRSA isolates from an infected wound in Gamo Gofa Zone, South Ethiopia, so that they would have been recommended as therapeutic agents after pharmaceutical standardization and clinical trials.

## 2. Materials and Methods

### 2.1. Study Design, Area, and Period

An experimental study design was conducted at Arba Minch University Medical Microbiology and Parasitology Laboratory from May 2017 to November 2017.

### 2.2. Sample Size Determination and Sampling Technique

The sample size was determined using sample size determination for estimation of single population proportion formula. Taking 97% prevalence of multiple drug resistance (MDR) isolates from previous study [25], 95% confidence interval (*z* = 1.96), and 5% marginal error (*d* = 0.05) the initial sample size is

(1) $$\begin{array}{c} n=\frac{{\left(z_{\propto /2}\right)}^{2}\;*\;P\left(1-P\right)}{d^{2}}=\frac{{\left(1.96\right)}^{2}\;*\;0.97\;*\;0.03}{{\left(0.05\right)}^{2}}\approx 45. \end{array}$$

Finally, by considering a 10% (≈5 subjects) nonresponse rate, the final sample size was determined as *n*+5 ≈ 50.

Regarding sampling technique, all isolated MRSA were included in the experiment.

### 2.3. Wound Sampling Procedure

Open and clinically infected wound swabs were aseptically obtained after the wound was cleansed with sterile normal saline and collected by trained nurses. The specimen was collected on sterile cotton swab by rotating with sufficient pressure. The samples were transported to the laboratory after collection using Amies transport media.

### 2.4. Culture and Identification

Swabs collected were streaked on blood agar and mannitol salt agar (Oxoid) by using a sterile inoculation loop. The plates were incubated at 35–37°C for 24–48 hours. Preliminary identification of bacteria was based on colony characteristics of the organisms, such as hemolysis on blood agar, changes in physical appearance in differential media, and enzyme activities of the organisms. Isolates were identified based on their gram reaction, catalase, and coagulase test results.

### 2.5. Antibacterial Susceptibility Testing

Susceptibility testing was performed by Kirby–Bauer disk diffusion technique according to criteria set by Clinical Laboratory Standard Institute (CLSI), 2016. The inoculums were prepared and suspended in sterile normal saline. The density of suspension was determined by comparison with opacity standard on McFarland 0.5 barium sulphate solution. The test organism was uniformly seeded over the Mueller–Hinton agar (Oxoid) surface and exposed to the concentration gradient of the antibiotic followed by incubation at 37°C for 16–18 hours. Diameters of the zone of inhibition around the discs were measured to the nearest millimeter using a ruler and classified as sensitive, intermediate, and resistant according to the standardized table supplied by CLSI, 2016. The antibiotics tested were ciprofloxacin (5 *μ*g), gentamicin (10 *μ*g), tetracycline (30 *μ*g), co-trimoxazole (25 *μ*g), chloramphenicol (30 *μ*g), amikacin (30 *μ*g), clindamycin (10 *μ*g), erythromycin (15 *μ*g), and vancomycin (30 *μ*g). These antimicrobials were selected based on the availability and prescription frequency of these drugs in the study area.

### 2.6. Test Organisms

All screened methicillin-resistant *S*. *aureus* from the wound was used. Methicillin-resistant *S*. *aureus* was identified phenotypically based on its resistance to oxacillin (1 *μ*g) and cefoxitin (30 *μ*g) by the disc diffusion method performed on modified Muller–Hinton agar (Oxoid, Basingstoke, UK). Based on the CLSI, 2016 guideline, the zone of inhibition is interpreted and grouped into methicillin-sensitive and methicillin-resistant *S*. *aureus* [18].

### 2.7. Honey Sample

Four kinds of honey were harvested from beekeepers of the Gamo Gofa zone using purposive sampling technique. Honey was collected in sterile screwed cups/culture bottle. Each honey sample was first filtered with a sterile mesh/gauze to remove debris and then streaked on blood agar plate to check sterility and stored at 2–8°C until used.

### 2.8. Preparation of Honey Solutions

Hundred percent pure honey (100% v/v) was obtained after filtered using sterile gauze. To get 75% honey solutions (v/v), 0.75 ml of honey was diluted in 0.25 ml sterilized distilled water. Further serial dilutions of 0.5 ml of each and 0.25 ml of honey and 0.75 ml of sterile distilled water were added to obtain 50% and 25% honey solutions (v/v), respectively.

### 2.9. Susceptibility Testing of Honey

Susceptibility testing was performed by Kirby–Bauer disk diffusion technique according to criteria set by CLSI, 2016. The inoculums were prepared by picking parts of similar test organisms with a sterile wire loop and suspended in sterile normal saline. The density of suspension was determined by comparison with opacity standard on McFarland 0.5 barium sulphate solution. A sterile swab was dipped into the suspension of the isolate, squeezed free from excess fluid against the side of the tube, and then spread over the agar plate. The test organism was uniformly seeded over the Mueller–Hinton agar (Oxoid) surface, and the plates were left on the bench for the excess fluid being absorbed. Using a sterile cork borer (6 mm diameter, 4 mm deep, and about 2 cm apart), wells were made in the agar medium. Using a micropipette, 50 *µ*L of honey with the concentration of 75%, 50%, and 25% was added to the wells in the plate. The plates were incubated at 37°C for 24 h. The mean diameters of inhibition zones were measured in mm, and the results were recorded. A positive control well was equally filled with vancomycin (30 *µ*g), while sterile distilled water used as negative control. The experiment was repeated 3× for each strain.

### 2.10. Determination of Minimum Inhibitory Concentration

Minimum inhibitory concentration (MIC) and minimum bactericidal concentration (MBC) of the antimicrobial agents were determined for each isolate by the tube dilution method. Briefly, ten sterile test tubes were placed in the rack, labeled each 1 through 8. Honey control tubes (HC), broth control tube (BC), and growth control tube (GC) were used as quality controls. One milliliter of freshly prepared nutrient broth was added to each tube, sterilized, and cooled. Then one milliliter of undiluted honey solution 100% was added to test tube number 1 and HC with a sterile micropipette and tips. Then, twofold serial dilution was performed by transferring 1 ml undiluted honey into the second tube with separate sterile micropipette and tips and vortexed for homogenization. After a through mixing, 1 ml was transferred with another sterile micropipette from tube 2 and tube 3. These procedures continued until the eighth tube with a dilution of 1 : 128 was reached, and finally 1 ml was taken and discarded from tube 8. The GC tube that received no honey and BC that received no bacterial inoculums served as growth control while the HC tube that received no bacterial inoculums served as a honey control. Except for the HC tube, each tube was inoculated with 1 ml of the culture of the respective prepared organism. The whole procedures were repeated for all the organisms tested to each of the honey. Tubes were then incubated at 37°C for 24 h and observed by visual inspections for the presence and absence of growth (turbidity). MIC was recorded as the lowest concentration of honey that inhibited bacterial growth (no visible growth or turbidity).

### 2.11. Determination of Minimum Bactericidal Concentration

To determine the MBC, incubated tubes, showing no visible sign of growth/turbidity in MIC, were subcultured onto sterile nutrient agar plates by the streak plate method and incubated at 37°C for 24 h aerobically. The least concentration of honey that did not show growth of test organisms was considered as the MBC. Then inoculated plates were scored as bactericidal if no growth, bacteriostatic if there is light to moderate growth, and no antibacterial activity if there is heavy growth.

### 2.12. Data Quality Control

Data quality was ensured at various activities of the study (preanalytical, analytical, and postanalytical) by following prepared standard operating procedure (SOP). Culture media was prepared according to the manufacturer's instruction, and the sterility was checked by incubating representative of the batch at 35–37°C overnight and observing bacterial growth. Those batch of the media showed growth was discarded. A control strain of *S*. *aureus* (ATCC-25923) was used to check the quality of the media and potency of the antibiotics used for positive controls. Pretesting of the questionnaires was done on about 5% of the total respondents, and the completed questionnaires were checked, and corrections were made on a daily base.

## 3. Results

### 3.1. Study Population and Patient Characteristics

A total of 50 samples collected from patients with clinical evidence of wound infection (patients with complaints of discharge, pain, swelling, foul smelling, and chronic wound) from May to November 2017. The subjects included 25 (50%) males and females, respectively. The incidence of the wound was highest among the age group 16–30 years with 25 (50%), followed by age group ≥ 15 years, 11 (22%), among the total number of patients studied. Housewives and students (15 (30%)) had the highest infection rate among the occupational group followed by government employees (8 (16%)). The sociodemographic characteristics of the participants are summarized in (Table 1).

### 3.2. Prevalence of MRSA

**Figure 1:**
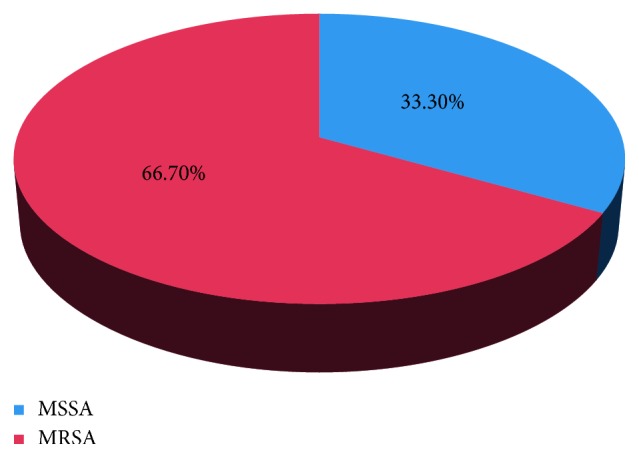
Prevalence of MRSA and MSSA among patients with infected wounds at Arba Minch Hospital, Arba Minch, May–November 2017.

As stated in Figure 1, among isolated *S*. *aureus* (15 (41.7%)) screened for methicillin resistance, MRSA accounts 10 (66.7%), while the remaining 5 (33.3%) were methicillin-sensitive *S*. *aureus* (MSSA).

### 3.3. Antibiotic Susceptibility Pattern

Methicillin-resistant clinical isolates were tested against selected 9 antibiotics that prescribed in the area and recommended by the Clinical Laboratory Standard. Susceptibility of pathogens to tested antibiotics was varied. Eighty percent of pathogens were resistant to tetracycline followed by 40% co-trimoxazole and 30% erythromycin. However, all isolates showed high sensitivity to vancomycin, amikacin, ciprofloxacin, and gentamicin (Table 2).

### 3.4. Disk Diffusion Honey Sensitivity Test

**Figure 2:**
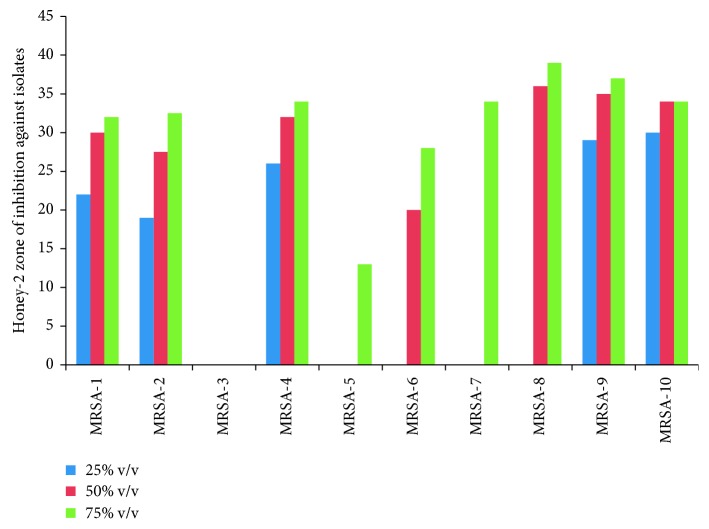
Susceptibility of MRSA to honey-2 at 25% v/v, 50% v/v, and 75% v/v collected from Arba Minch Province, Arba Minch, May–November 2017.

**Figure 3:**
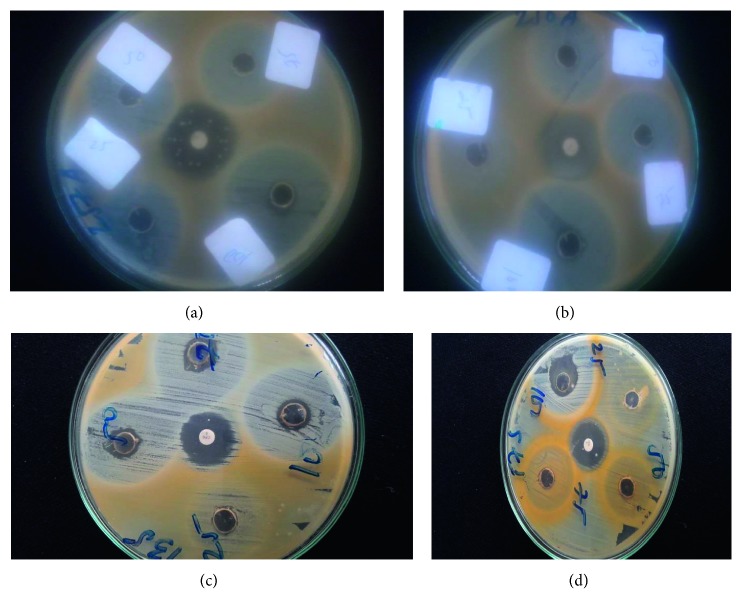
Disk diffusion susceptibility of MRSA to honey-2 at 25% v/v, 50% v/v, 75% v/v, and 100% v/v collected from Arba Minch Province, Arba Minch, May–November 2017.

In this study, four different varieties of honey labeled as honey-1 to -4 were collected and tested for their antimicrobial potential from Arba Minch Province on ten methicillin -resistant *S*. *aureus* (MRSA) clinical isolates. All collected honey samples show an antibacterial effect at 100% v/v while some of them showed a bactericidal and bacteriostatic effect at 75 and 50% v/v. In general, zones of inhibition ranged from 0–39 mm; accordingly, honey-2 at 75% v/v concentration showed the largest average zone of inhibition and selected for further minimum inhibitory concentration and minimum bactericidal concentration (Figures 2 and 3).

### 3.5. Determination of MIC

The MIC determination was done only for honey-2 because of the potent antibacterial effect is shown on disk diffusion during the preliminary sensitivity assay. The mean MICs of the honey-2 samples with clinical isolates of MRSA are presented in Table 3. The mean MIC of MRSA isolates ranged from 9.38 to 37.5% v/v, while most of the isolates 6 (60%) showed MIC at 18.75% v/v and the least 1 (10%) at 37.5% v/v.

### 3.6. Determination of MBC

**Figure 4:**
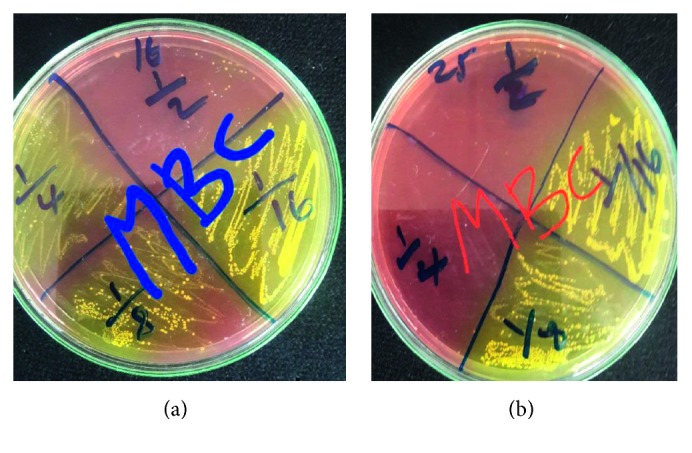
Minimum bactericidal determination against MRSA isolates from Arba Minch Province, Arba Minch, May–November 2017.

As mentioned above, the most active honey was further assayed to determine its minimum inhibitory concentration and minimum bactericidal concentration against 10 MRSA isolates. Consequently, honey-2 at 75% v/v was selected. The MBC ranged from 9.38 to 37.5 (Table 4 and Figure 4). Partial inhibition of 50% of the test MRSA was observed starting from 18.75% v/v, and 100% complete inhibition was observed at 37.5% v/v of honey. Hence, the MBC value of 30% of tested microorganisms was found to be similar to the MIC value of tested organisms at 18.75% v/v.

## 4. Discussion

Wound infections have been a problem in the field of medicine for a long time, and the problem complicated more recently because of increased antimicrobial resistance. This is a problem too for public, researchers, clinicians, and drug companies looking for effective drugs. Therefore, antimicrobial resistance may increase complications and costs associated with procedures and treatment that leads to a continued search for new agents [26].

*S*. *aureus* is a Gram-positive bacterium which is a major pathogen implicated in skin infections such as impetigo, furuncles, boils, sties, pustules, burns, and wounds. Antibiotic-resistant strains of *S*. *aureus* are the major cause of infections especially in a hospital setting [27]. Strains of *S*. *aureus* that were fully sensitive to penicillin now developed resistance to methicillin, and other latest ones resort antibiotics too [28].

In this study, a total of 50 patients suffering from wound infections were included, of which male and female each accounts 25 (50%), respectively. The incidence of wound infection was relatively more common in males (68%) than in females (64%). This is in agreement with studies performed in different parts of Ethiopia like Bahirdar [29], Addis Ababa [30], and Gondar [31] Nigeria [32, 33], and India [34]. This slight differences might be explained by the fact that traditionally, in this country, mainly males are involved in occupations such as farming, construction works, transportation, and industry works where the likely exposure to trauma is common.

*Staphylococcus aureus* has been known to acquire resistance to most antibiotics including the penicillinase-resistant ones like methicillin. A study carried out in USA [35] found an incidence of 20.6% MRSA and 10% in [36] and 21.7% in [37], which are more lower when compared with that in the current study (66.7%); in contrast to this, higher incidences of 45% and 58.2% MRSA have been documented by Eagye et al. [38] and Keith et al. [39]. As well higher incidence of (63.4%) was reported in China [40], which is similar to the current study. We found that all the MRSA strains were (100%) sensitive to vancomycin and amikacin, followed by (90%) gentamicin and ciprofloxacin, respectively. This finding could have relevant clinical use in the antibiotic policy guidelines for hospitals.

Honey antimicrobial properties will vary depending on the type of honey, geographical location, and flower from which the final product is derived [41]. Hence, the present study aimed to test the antimicrobial activity of honey against MRSA isolates from wound infection.

Hence, in the present study, all tested honey samples show an antibacterial effect against clinical isolates during disk diffusion technique. Especially it showed complete inhibitory effect with a clear zone of inhibition against the tested organism at 100% v/v. The bactericidal concentrations of honey against MRSA in our study were between 50 and 100%. This concentration was higher than the findings of other researchers [19, 42, 43].

Tested honey sample (honey-2) was found to have both bacteriostatic and bactericidal properties with different concentrations ranging from 9.38–37.5% v/v. Growth retardation and complete inhibition on 70% of the test organisms were observed at a concentration of 18.75% v/v of honey.

The MIC and MBC values in this study indicated that tested honey (honey-2) has potential bactericidal and bacteriostatic activities against multidrug-resistant clinical isolates of MRSA bacteria. This was similar to other studies conducted elsewhere [42, 44, 45].

The percentage by volume of honey to completely prevent the growth of MRSA was in the range of 18.75–37.5% v/v. In contrary to this, a study conducted in Ethiopia has shown that the percentage by volume of honey to completely prevent growth of *S*. *aureus* to be 6.5% v/v [42] which is lower concentration than our result. Another study by Willix has also found that the % (v/v) of Manuka honey to completely prevent growth for *S*. *aureus* was 1.8 [46]. This difference might be due to the difference in the species of bees and the differences in the test methods used and test organisms or might be due to the variation in antimicrobial activities of honey in different geographical locations in preparing their valuable honey (nectars and pollens).

Even though tested honey shows antibacterial effect, many studies have demonstrated that not all honey samples have the same degree of antibacterial activity. Therefore, the sensitivity of MRSA isolates cannot be compared using the results from different studies, as the honey used in the studies may have had widely differing antimicrobial activities. Therefore, the MIC and MBC values determined with the MRSA strains in this study indicate that there is no much difference in sensitivity (effectiveness of honey to inhibit growth or to kill the bacteria) to honey. Hence, honey has potential in the decontamination of wounds colonized antibiotic-resistant strains of bacteria like MRSA. This supports the existing local traditional practice of using honey to treat wound infections [47, 48].

## 5. Conclusions

Honey had antimicrobial properties (bacteriostatic and bactericidal activities) against MRSA organisms tested. The antibacterial potency of “honey-2” on the test organism of MRSA isolates was highly effective with MIC and MBC ranged from 9.38–37.5% v/v.

## Figures and Tables

**Table 1:** Wound infection and sociodemographic characteristics of the patients with wound infection at Arba Minch Hospital, Arba Minch, May–November 2017.

Demographic characters	Infected (no. (%))	Not infected (no. (%))	Total (no. (%))
*Sex*			
Male	17 (68)	8	25 (50)
Female	16 (64)	9	25 (50)
Total	33 (66)	17 (34)	50 (100)

*Age in years*			
≤15	5	6	11
16–30	20 (80)	5	25 (50)
31–44	4	4	8
45–59	4	2	6
Total	33 (66)	17 (34)	50 (100)

*Occupation*			
Students	12	3	15 (30)
Housewives	10	5	15 (30)
Civil servants	6	2	8
Self-employed	2	3	5
Merchants	2	2	4
Farmers	1	2	3
Total	33 (66)	17 (34)	50 (100)

*Educational status*			
Illiterate	9	7	16 (32)
Literate	24 (70.6)	10	34 (68)
Total	33 (66)	17 (34)	50 (100)

**Table 2:** Antimicrobial susceptibility pattern of MSSA and MRSA from infected wound patients at Arba Minch Hospital, Arba Minch, South Ethiopia, May–November 2017.

Isolate		Antimicrobial agents (no. (%))								
		VA	CLN	ERY	AK	TET	CHL	CPR	COT	GEN
MSSA (*n*=5)	S	5 (100)	5 (100)	4 (80)	5/100	2 (30)	3 (60)	4 (80)	4 (80)	5 (100)
	R	0	0	1 (20)	0	3 (60)	2 (30)	1 (20)	1 (20)	0

MRSA (*n*=10)	S	10 (100)	9 (90)	7 (70)	10/100	2 (20)	8 (80)	9 (90)	6 (60)	9 (90)
	R	0	1 (10)	3 (30)	0	8 (80)	2 (20)	1 (10)	4 (40)	1 (10)

Total (*n*=15)	S	15 (100)	14 (93.3)	11 (73.3)	15/100	4 (26.7)	11 (73.3)	13 (86.7)	10 (66.7)	14 (93.3)
	R	0	1 (6.7)	4 (26.7)	0	11 (73.3)	4 (26.7)	2 (13.3)	5 (33.3)	1 (6.7)

VA = vancomycin; CLN = clindamycin; ERY = erythromycin; AK = amikacin; TET = tetracycline; CHL = chloramphenicol; CPR = ciprofloxacin; COT = co-trimoxazole; GEN = gentamicin.

**Table 3:** MIC (% v/v) of honey-2 samples against MRSA isolates in Arba Minch Province, Arba Minch, May–November 2017.

Honey dilution									
Test bacteria	Net (1)	1/2	1/4	1/8	1/16	1/32	1/64	1/128	MIC (% v/v)
MRSA-1	−	−	−	+	+	+	+	+	18.75
MRSA-2	−	−	−	−	+	+	+	+	9.38
MRSA-3	−	−	−	+	+	+	+	+	18.75
MRSA-4	−	−	−	−	+	+	+	+	9.38
MRSA-5	−	−	−	−	+	+	+	+	9.38
MRSA-6	−	−	−	+	+	+	+	+	18.75
MRSA-7	−	−	−	+	+	+	+	+	18.75
MRSA-8	−	−	+	+	+	+	+	+	37.5
MRSA-9	−	−	−	+	+	+	+	+	18.75
MRSA-10	−	−	−	+	+	+	+	+	18.75

− , no growth (bactericidal); + , growth.

**Table 4:** MBC (% v/v) of honey-2 samples against MRSA isolates in Arba Minch Province, Arba Minch, May–November 2017.

Honey dilution							
Test bacteria	Net (1)	1/2	1/4	1/8	1/16	1/32	MBC (% v/v)
MRSA-1	−	−	+	++	+++	+++	37.5
MRSA-2	−	−	−	+	++	+++	18.75
MRSA-3	−	−	+	++	+++	+++	37.5
MRSA-4	−	−	−	+	++	+++	18.75
MRSA-5	−	−	−	+	++	+++	18.75
MRSA-6	−	−	+	++	++	+++	37.5
MRSA-7	−	−	−	+	++	+++	18.75
MRSA-8	−	−	+	++	+++	+++	37.5
MRSA-9	−	−	+	++	+++	+++	37.5
MRSA-10	−	−	−	+	++	+++	18.75

− , no growth (bactericidal);  + , light growth;  ++ , moderate growth (bacteriostatic);  +++ , heavy growth (no antibacterial potential).

## Data Availability

The data used to support the findings of this study are included within the article.

## Abbreviations

ATCC: American Type Culture Collection
BC: Broth control
CLSI: Clinical Laboratory Standard Institute
GC: Growth control
HC: Honey control
MBC: Minimum bactericidal concentration
MDR: Multiple drug resistance
MIC: Minimum inhibitory concentration
MRSA: Methicillin-resistant *Staphylococcus aureus*
MSSA: Methicillin-susceptible *Staphylococcus aureus*
SOP: Standard operating procedure
WHO: World Health Organization.

## References

[1] Maghsoudi H., Salehi F., Khosrowshahi M. K., Baghaei M., Nasirzadeh M., Shams R. (2011). Comparison between topical honey and mafenide acetate in treatment of burn wounds. *Annals of Burns and Fire Disasters*.

[2] Tan M. K., Adli D. S. H., Tumiran M. A., Abdulla M. A., Yusoff K. M. (2012). The efficacy of gelam honey dressing towards excisional wound healing. *Evidence-Based Complementary and Alternative Medicine*.

[3] Osho A., Bello O. (2010). Antimicrobial effect of honey produced by on some common human pathogens. *Asian Journal of Experimental Biological Science*.

[4] Davies J. (1994). Inactivation of antibiotics and the dissemination of resistance genes. *Science*.

[5] Pieters L., Vlietinck A. J. (2005). Bioguided isolation of pharmacologically active plant components, still a valuable strategy for the finding of new lead compounds?. *Journal of Ethnopharmacology*.

[6] Balunas M. J., Kinghorn A. D. (2005). Drug discovery from medicinal plants. *Life Sciences*.

[7] Kwakman P., De Boer L., Ruyter-Spira C. (2011). Medical-grade honey enriched with antimicrobial peptides has enhanced activity against antibiotic-resistant pathogens. *European Journal of Clinical Microbiology & Infectious Diseases*.

[8] Shears P. (2000). Antimicrobial resistance in the tropics. *Tropical Doctor*.

[9] Mulu A., Tessema B., Derbie F. (2005). In vitro assessment of the antimicrobial potential of honey on common human pathogens. *Ethiopian Journal of Health Development*.

[10] Bansal V., Medhi B., Pandhi P. (2005). Honey–a remedy rediscovered and its therapeutic utility. *Kathmandu University Medical Journal*.

[11] Omoya F., Akharaiyi F. (2011). Mixture of honey and ginger extract for antibacterial assessment on some clinical isolates. *International Journal of Pharmaceutical and Biomedical Research*.

[12] Dixon B. (2003). Bacteria can’t resist honey. *Lancet Infectious Diseases*.

[13] Mohapatra D., Thakur V., Brar S. (2011). Antibacterial efficacy of raw and processed honey. *Biotechnology Research International*.

[14] Henriques A., Jackson S., Cooper R., Burton N. (2006). Free radical production and quenching in honeys with wound healing potential. *Journal of Antimicrobial Chemotherapy*.

[15] Sharp A. (2009). Beneficial effects of honey dressings in wound management. *Nursing Standard*.

[16] White J., Doner L. W. (1980). Honey composition and properties. *Beekeeping in the United States Agriculture Handbook*.

[17] Olaitan P. B., Adeleke O. E., Ola I. O. (2007). Honey: a reservoir for microorganisms and an inhibitory agent for microbes. *African Health Sciences*.

[18] Lusby P., Coombes A., Wilkinson J. (2002). Honey: a potent agent for wound healing?. *Journal of WOCN*.

[19] Molan P. C. (1992). The antibacterial activity of honey. *Bee World*.

[20] Pieper B. (2009). Honey-based dressings and wound care. *Journal of Wound, Ostomy and Continence Nursing*.

[21] White R., Molan P., White R., Cooper R., Molan P. (2005). A summary of published clinical research on honey in wound management. *Honey: A Modern Wound Management Product*.

[22] Molan P. C. (1999). The role of honey in the management of wounds. *Journal of Wound Care*.

[23] Zhang X. (2000). *General Guidelines for Methodologies on Research and Evaluation of Traditional Medicine*.

[24] Kassaye K. D., Amberbir A., Getachew B., Mussema Y. (2007). A historical overview of traditional medicine practices and policy in Ethiopia. *Ethiopian Journal of Health Development*.

[25] Mama M., Abdissa A., Sewunet T. (2014). Antimicrobial susceptibility pattern of bacterial isolates from wound infection and their sensitivity to alternative topical agents at Jimma University Specialized Hospital, South-West Ethiopia. *Annals of Clinical Microbiology and Antimicrobials*.

[26] Sule A. M., Thanni L. O. A., Odu S. O. A., Olusanya O. (2002). Bacterial pathogens associated with infected wounds in Ogun State University Teaching Hospital, Sagamu, Nigeria. *African Journal of Clinical and Experimental Microbiology*.

[27] Mudey A. B., Kesharwani N., Mudey G. A., Goyal R. C., Dawale A. K., Wagh V. V. (2010). Health status and personal hygiene among food handlers working at food establishment around a rural teaching hospital in Wardha District of Maharashtra, India. *Global Journal of Health Science*.

[28] Naik D., Teclu A. (2010). A study on antimicrobial susceptibility pattern in clinical isolates of *Staphylococcus aureus* in Eritrea. *Pan African Medical Journal*.

[29] Biadglegne F., Abera B., Alem A., Anagaw B. (2009). Bacterial isolates from wound infection and their antimicrobial susceptibility pattern in Felege Hiwot Referral Hospital, North West Ethiopia. *Ethiopian Journal of Health Sciences*.

[30] Tekie K K. (2008). *Surgical wound infection in Tikur Anbessa Hospital with special emphasis on Pseudomonas aeruginosa*.

[31] Gelaw A. (2011). *Isolation of bacterial pathogens from patients with postoperative surgical site infections and possible sources of infections at University of Gondar Hospital*.

[32] Ohalete C. N., Obi R. K., EmeaKoroha M. C. (2012). Bacteriology of different wound infection and their antimicrobial susceptibility patterns in Imo state Nigeria. *World Journal of Pharmaceutical Sciences*.

[33] Amoran O. E., Sogebi A. O., Fatugase O. M. (2014). Rates and risk factors associated with surgical site infections in a tertiary care center in South-Western Nigeria. *International Journal of Tropical Disease & Health*.

[34] Goswami N., Goswami A. P., Tripathi C., Trivedi H., Patel T. (2011). Antibiotic sensitivity profile of bacterial pathogens in postoperative wound infections at a tertiary care hospital in Gujarat, India. *Journal of Pharmacology and Pharmacotherapeutics*.

[35] Weigelt J. A., Lipsky B. A., Tabak Y. P., Derby K. G., Kim M., Gupta V. (2010). Sugical site infections: causative pathogens and associated outcomes. *American Journal of Infection Control*.

[36] Aggarwal A., Khanna S., Arora U., Devi P. (2001). Correlation of β-lactamase production/methicillin resistance and phage pattern of *Staphylococcus aureus*. *Indian Journal of Medical Sciences*.

[37] Kownhar H., Shankar E. M., Vignesh R., Sekar R., Velu V., Rao U. A. (2008). High isolation rate of *Staphylococcus aureus* from surgical site infections in an Indian hospital. *Journal of Antimicrobial Chemotherapy*.

[38] Eagye K. J., Kim A., Laohavaleeson S., Kuti J. L., Nicolau D. P. (2009). Surgical site infections: does inadequate antibiotic therapy affect patient outcomes?. *Surgical Infections*.

[39] Keith S. K., Anderson D. J., Sloane R. (2009). The effect of surgical site infection on older operative patients. *Journal of the American Geriatrics Society*.

[40] Wang S.-H., Sun Z.-L., Guo Y.-J. (2010). Meticillin-resistant *Staphylococcus aureus* isolated from foot ulcers in diabetic patients in a Chinese care hospital: risk factors for infection and prevalence. *Journal of Medical Microbiology*.

[41] Sherlock O., Dolan A., Athman R. (2010). Comparison of the antimicrobial activity of ulmo honey from Chile and manuka honey against methicillin-resistant *Staphylococcus aureus*, *Escherichia coli* and *Pseudomonas aeruginosa*. *BMC Complementary and Alternative Medicine*.

[42] Ahmed M., Sahile S., Subramanian C. (2014). Evaluation of antibacterial potential of honey against some common human pathogens in North Gondar Zone of Ethiopia. *International Journal of Pure and Applied Zoology*.

[43] Molan P. C., Julie B. (2000). Using honey dressings: the practical considerations. *Nursing Times*.

[44] Allen K. L., Hutchinson G., Molan P. C. The potential for using honey to treat wounds infected with MRSA and VRE.

[45] Getaneh A., Belyhun Y., Moges F. (2013). In vitro assessment of the antimicrobial efect of Ethiopian multi-flora honey on methicillin resistant *Staphylococcus aureus*. *International Journal of Current Research and Academic Review*.

[46] Willix D. J., Molan P. C., Harfoot C. G. (1992). A comparison of the sensitivity of wound-infecting species of bacteria to the antibacterial activity of manuka honey and other honey. *Journal of Applied Bacteriology*.

[47] AL-Haj N. A., Amghalia E., Shamsudin M. N., Abdullah R., Mohamed R., Sekaw Z. (2009). Antibacterial activity of honey against methicillin-resistant *Staphylococcus aureus*. *Research Journal of Biological Sciences*.

[48] George N. M., Cutting K. F. (2007). Antibacterial Honey: in vitro activity against clinical isolates of MRSA, VRE, and other multi-resistant Gram-negative organisms including *Pseudomonas aeruginosa*. *Wounds*.

